# Changes in moderately low birthweight infant feeding, care, and health outcomes before compared to during the COVID-19 pandemic in Malawi

**DOI:** 10.7189/jogh.13.06025

**Published:** 2023-06-30

**Authors:** Friday Saidi, Rana R Mokhtar, Irving F Hoffman, Melda Phiri, Fadire Nyirenda, Kingsly Msimuko, Msandeni Chiume, Linda Vesel, Katherine EA Semrau, Tisungane Mvalo

**Affiliations:** 1 UNC Project Malawi, Lilongwe, Malawi; 2 Department of Obstetrics and Gynecology, Kamuzu University of Health Sciences, Kamuzu Central Hospital Campus, Lilongwe, Malawi; 3 Department of Obstetrics and Gynecology, School of Medicine, University of North Carolina at Chapel Hill, North Carolina, USA; 4 Ariadne Labs, Harvard T.H. Chan School of Public Health / Brigham and Women’s Hospital, Boston, Massachusetts, USA; 5 Institute for Global Health and Infectious Diseases, University of North Carolina at Chapel Hill School of Medicine, Chapel Hill, North Carolina, USA; 6 Department of Medicine, Harvard Medical School, Boston, Massachusetts, USA; 7 Department of Pediatrics, Kamuzu Central Hospital, Ministry of Health, Malawi; 8 Department of Pediatrics, University of North Carolina at Chapel Hill School of Medicine, Chapel Hill, North Carolina, USA

## Abstract

**Background:**

The coronavirus disease 2019 (COVID-19) and the measures taken to minimise its spread have significantly impacted mother- and infant-related healthcare. We describe the changes in newborn feeding, lactation support, and growth outcomes before compared to during the COVID-19 pandemic among moderately low birthweight infants (LBW) (1.5 to <2.5kg) in Malawi.

**Methods:**

The data presented here are part of the Low Birthweight Infant Feeding Exploration (LIFE) study, a formative, multisite, mixed methods observational cohort study. In this analysis, we included infants born at two public hospitals in Lilongwe, Malawi between 18 October 2019 and 29 July 2020. We categorised births as “pre-COVID-19 period” (before 1 April 2020) and “during COVID-19 period” (on or after 2 April 2020) and used descriptive statistics and mixed effects models to examine differences in birth complications, lactation support, feeding, and growth outcomes between the two time periods.

**Results:**

We included 300 infants and their mothers (n = 273) in the analysis. Most infants (n = 240) were born during the pre-COVID-19 period; 60 were born during the pandemic period. The latter group had a lower prevalence of uncomplicated births (35.8%) compared to pre-pandemic period group (16.7%) (*P* = 0.004). Fewer mothers reported early initiation of breastfeeding in the pandemic period (27.2%) compared to the pre-pandemic period (14.6%) (*P* = 0.053), along with significantly less breastfeeding support, particularly in view of discussion of proper latching (44.9% during COVID-19 vs 72.7% pre-COVID-19; *P* < 0.001) and physical support with positioning (14.3% vs 45.5% pre-COVID-19 *P* < 0.001). At 10 weeks of age, the prevalence of stunting was 51.0% pre-COVID-19 vs 45.1% during COVID-19 (*P* = 0.46), the prevalence of underweight was 22.5% pre-COVID-19 vs 30.4% during COVID-19 (*P* = 0.27), and the prevalence of wasting was 0% pre-COVID-19 vs 2.5% during COVID-19 (*P* = 0.27).

**Conclusions:**

Our findings highlight the continued need to optimise early initiation of breastfeeding and lactation support for infants during COVID-19 and future pandemics. More studies are needed to evaluate the long-term outcomes of moderately LBW born during the COVID-19 pandemic (including growth outcomes) and determine the impact of restrictive measures on access to lactation support and promotion of early initiation of breastfeeding.

The coronavirus disease 2019 (COVID-19) and the non-pharmacological measures taken to mitigate its spread have significantly impacted maternal- and child-related healthcare [1,2], threatening progress in preventing maternal and child mortality, particularly in sub-Saharan Africa, where mortality among these population groups is already disproportionately high [3]. Despite advancements in reducing maternal and child mortality since the inception of the Sustainable Development Goals (SDGs) in 2015 [4], many low- and middle-income countries (LMICs) are still not on track to achieve the proposed targets by 2030 [3]. In fact, modelling studies are predicting a possible reversal in the already achieved gains due to the COVID-19 pandemic [5,6].

Despite low COVID-19-related mortality rates among children and women of reproductive age [7,8], the pandemic has disproportionately affected access to high-quality health services for these populations due to movement restrictions, physical distancing measures, rationing of healthcare resources, and the paucity of healthcare providers. The restrictions in movement were dictated by both governmental regulations and by individuals being apprehensive to present to a facility for fear of being infected with COVID-19. In LMICs like Malawi, managing the pandemic alongside pre-existing health priorities amid an already weakened health system has proven challenging. This is comparable to the 2014 Ebola virus outbreak, which adversely affected maternal and perinatal healthcare, despite low morbidity and mortality caused directly by the disease [9,10].

Malawi’s first COVID-19 cases were reported on 2 April 2020 [11]. As of 2 January 2023, there were 647 991 suspected cases, 88 366 confirmed cases, and 2686 deaths [12]. In response to the pandemic, Malawi adopted several policies and restrictions that shifted resources away from maternal and child healthcare services and towards fighting the pandemic [13-15]. Healthcare experiences and care-seeking behaviours of mothers and children during childbirth and the postnatal period changed during the pandemic, which impacted the quality and quantity of maternal and newborn care, especially evidence-based, respectful care practices (such as having a guardian or birth companion in the labour ward), family visitations, zero separation between mothers and newborns, and promotion of early breastfeeding [16]. The COVID-19 pandemic also resulted in staff shortages due to illness and the 14-day isolation periods for exposed individuals. The deficits in quality of care delivery were drastic among preterm (<37 weeks gestation) and low birth weight (LBW) (<2.5kg) infants, who are already vulnerable populations in Malawi with increased nutritional risk and breastfeeding challenges [17].

The COVID-19 pandemic offers lessons in preparing for future outbreaks, disasters, COVID-19 waves, and other pandemics that may disrupt healthcare delivery to mother-infant dyads. The Low Birthweight Infant Feeding Exploration (LIFE) study, a large mixed methods observational cohort study, aimed to understand current feeding practices, growth patterns, and other health outcomes among moderately LBW infants (1.5 to <2.5 kg) born in 12 secondary and tertiary facilities in India, Malawi, and Tanzania; the study methods are already described elsewhere [18]. In this analysis, we used a subset of the study data from the Malawi site. We hypothesised that the COVID-19 pandemic had an adverse impact on newborn care, feeding, and growth in the first 10 weeks of life for infants born and cared for “during” compared to those “before” the COVID-19 pandemic in Malawi. We aimed to evaluate whether the COVID-19 pandemic adversely impacted early initiation of breastfeeding and lactation support in the early postnatal period among moderately LBW infants and to examine if infants born during the pandemic had lower anthropometric measurements during early infancy compared to those born pre-pandemic.

## METHODS

### Study design

The results presented here are part of the larger LIFE study, a formative, multisite, observational cohort study involving 12 facilities in India, Malawi and Tanzania using a convergent parallel, mixed-methods design [18]. We used quantitative data collected from one cohort of this study [19]. In this secondary analysis, we examine a portion of the data collected from mother-infant dyads in Malawi using maternal reports, patient chart reviews, and observations within 72 hours of birth (baseline) and at weeks 1, 2, 4, 6 and 10 of age. We categorised births before 1 April 2020 as “pre-COVID-19 period” and those on or after 2 April 2020 as “during COVID-19 period” and were able to compare them in relation to the data collection period in Malawi during the LIFE study. In this analysis, we followed-up the infants from baseline to week 10 of life, with the last visits occurring between 21 October 2019 and 30 July 2020.

### Study setting

We conducted this study at Kamuzu Central Hospital (KCH) and Bwaila District Hospital in Lilongwe, the capital of Malawi. Both facilities are operated by the Malawi Ministry of Health and serve a population of about 1.2 million in the main city and surrounding villages [20]. KCH is the only tertiary referral hospital in the central region of Malawi serving nine referral district hospitals; its maternity unit conducts approximately 4000 deliveries per year. Bwaila District Hospital is a secondary level hospital and has the most active maternity unit in the Central Region of Malawi, with approximately 18 000 deliveries annually.

### Study population

We screened mother-infant dyads for eligibility, including infants born with moderately LBW, living within 50km of the designated enrolment facility, born without congenital abnormalities that may impact feeding, born to legally adult mothers (>18 years if unmarried or >16 years if married), and surviving the first 72 hours after birth. We did not include infants whose mothers did not consent or died within 72 hours of life.

### Data collection

We captured data on feeding (lactation support, early initiation of breastfeeding, and feeding profile), infant growth (weight, length, head circumference, mid-upper arm circumference), length of stay, mortality, and neonatal intensive care unit (NICU) admission. We measured infant anthropometrics in triplicate using standardised calibrated equipment, including a mobile digital baby scale (Seca 334), infant measuring board (Seca 417), Shorr MUAC tape (WM-MUAC26), and Shorr 65 cm head circumference tape (SKU WM-S Tape) [20]. We used this data to calculate mean length-for-age z-score (LAZ), weight-for-age z-score (WAZ), and weight-for-length z-score (WLZ) using the International Fetal and Newborn Growth Consortium for the 21st Century (INTERGROWTH-21st) [21] growth standards for preterm infants and the World Health Organization (WHO) child growth standards for term infants [22].

### Definition of key variables

The primary exposure in this analysis was being born during the pre-COVID-19 period compared to during the pandemic period, as defined above. Primary growth outcomes included mean LAZ, WAZ, and WLZ. We also classified infants as stunted (LAZ less than -2), underweight (WAZ less than -2), and wasted (WLZ less than -2). We stratified infants into four LBW types at birth based on their gestational age (preterm: <37 weeks or term: ≥37 weeks) and size-for-gestational age (small-for-gestational age (SGA: <10 percentile of weight for gestational age), appropriate-for-gestational age (AGA: 10-90th percentile) and large-for-gestational age (LGA: >90th percentile)): preterm-SGA, preterm-AGA, preterm-LGA, and term-SGA. Feeding outcomes included early initiation of breastfeeding (placing infant to the breast <1 hour postpartum), mean timing of initiation of breastfeeding, and feeding profile at 10 weeks. We defined feeding profile as exclusive breastfeeding (EBF) (direct breastfeeding or expressed breastmilk feeding while allowing for provision of oral rehydration solution drops, and syrups as vitamins and medicines) [23], mixed milk feeding (breastmilk feeding plus formula), no breastmilk feeding (i.e. formula only), or no foods given if mothers breastmilk had not come in yet. We defined mother-infant separation as the time the mother and infant were not sharing a room during the facility stay. Other outcomes included length of stay in the hospital, hospitalisation, and mortality during the 10 weeks follow-up period.

### Data analysis

We performed descriptive analyses to capture frequencies, means, medians, and standard deviations of key maternal and infant characteristics and subsequent care and feeding indicators. We used χ^2^ tests to assess differences between pre-COVID-19 and during COVID-19 periods for binary variables, Wilcoxon rank sum tests to test for differences between the medians, and *t*-tests to calculate differences between the means. We assessed the association between period of birth (pre-pandemic vs during pandemic period) and growth outcomes (mean LAZ, WAZ, WLZ) at 10 weeks. We used an interaction term between time period of birth and visit week to assess the statistical significance of differences in growth over time. The models used a compound symmetry working correlation matrix to account for correlations for multiple births, were clustered by mother to account for twins, and were adjusted for potential confounders, including maternal education, maternal age, parity, wealth quintile, place of residence, infant sex, birth count, LBW type, and study site. We performed all analyses using SAS statistical software (SAS Institute Cary, NC, version 9.3). Details for the full LIFE study protocol are available elsewhere [18].

### Ethics

The Harvard TH Chan School of Public Health (IRB 10-0282), the Malawi National Health Science Research Committee (NHSRC2019/Protocol 19/03/2250), and the University of North Carolina at Chapel Hill Institutional Review Board (21905) granted the ethical approval for this study. We obtained written informed consent from each mother-infant dyad prior to study enrolment or data collection. We registered this study in clinicaltrials.gov (NCT04002908).

## RESULTS

### Maternal and infant characteristics

**Figure 1:**
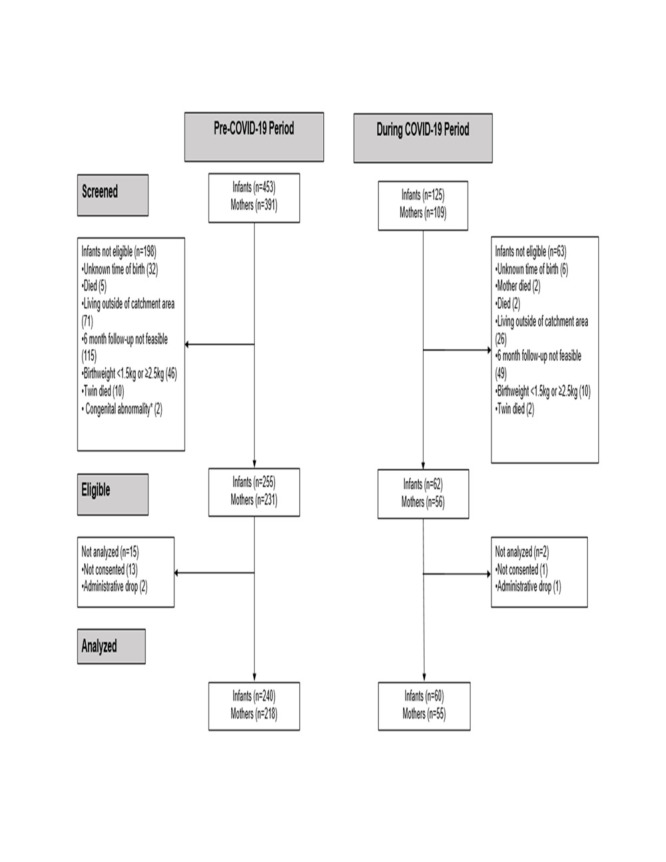
Flowchart of participants enrolled in the LIFE study prospective cohort in Malawi. Exclusion criteria for enrolment are not mutually exclusive. *Congenital abnormalities interfering with feeding.

**Figure 2:**
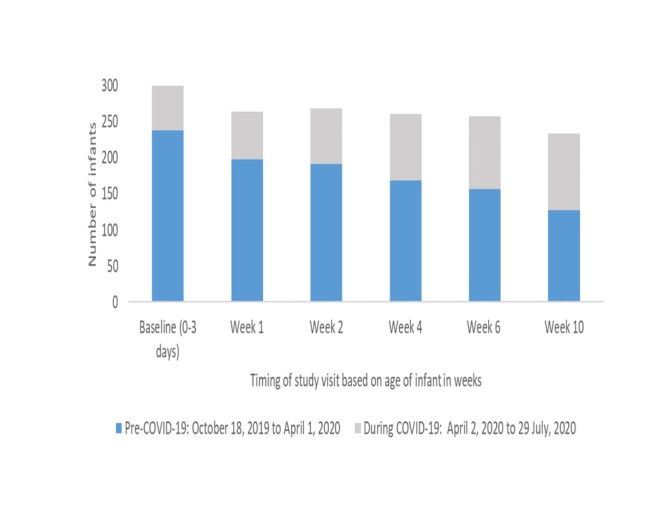
Number of infants in the LIFE study pre COVID-19 and during COVID-19. *Number of infants decreased over time due to missed visits, deaths and withdrawals at each study visit.

We screened 578 infants and 500 mothers for eligibility in Malawi; we excluded 261 (45.2%) infants as they did not meet the inclusion criteria (**Figure 1**). We analysed the data for 300 infants and their mothers (n = 272). Data collection took place between 13 September 2019 and 28 July 2021. Due to the COVID-19 pandemic, enrolment was temporarily paused from April 2020 to June 2020. **Figure 2** shows the number of infants followed up from birth to 10 weeks in the two groups (pre-COVID-19 period: n = 240, COVID-19 period: n = 60).

The enrolled mothers had a mean age of 25.3 years and were primarily living in urban areas (77.3%), married (93%), attended antenatal care (ANC) (98.9%), delivered singleton pregnancies (81.3%), and attained some primary education (56.8%) (**Table 1**). The HIV prevalence among the pooled population was 12.1%. Mothers enrolled during the pandemic were less likely to be HIV positive than those enrolled before the pandemic (7.3% during COVID-19 vs 13.3% pre-COVID-19). We found no differences in the ANC attendance before and during the pandemic period. Infant birth complications were present at higher rates during COVID-19 than pre-COVID-19.

**Table 1:** Maternal and infant baseline characteristics of participants enrolled before compared to during the COVID-19 pandemic in Malawi*

	Pre-COVID-19†	During COVID-19‡	*P*-value
**Maternal characteristics**	**n = 218**	**n = 55**	
Maternal age in years, mean (SD (range))	25.3 (6.1 (17.0-43.0))	24.6 (6.3 (17.0-45.0))	0.44
Marital status – married	202 (92.7)	52 (94.6)	0.63
Maternal education			
*Primary or less*	129 (59.2)	26 (47.3)	0.11
*Secondary or more*	89 (40.8)	29 (52.7)	
Place of residence			
*Rural*	51 (23.4)	11 (20.0)	0.59
*Urban*	167 (76.6)	44 (80.0)	
Any antenatal care – visit attendance	216 (99.1)	54 (98.2)	0.49
Mother’s parity			
*1*	26 (47.3)	78 (35.8)	0.09
*2*	14 (25.5)	45 (20.6)	
*≥3*	15 (27.3)	95 (43.6)	
Number babies in delivery			
*Singleton*	175 (80.3)	47(85.5)	0.38
*Twins*	43 (19.7)	8 (14.5)	
HIV positive	29 (13.3)	4 (7.3)	0.09
Maternal complications			
*No complication*	180 (82.6)	45 (81.8)	0.90
*Fever*	1 (0.46%)	0 (0)	0.61
*Heavy bleeding*	6 (2.75)	0 (0)	0.21
*High blood pressure*	15 (6.9)	4 (7.3)	0.92
*Convulsions*	2 (0.92)	0	0.48
**Infant characteristics**	**n = 240**	**n = 60**	
Female	127 (52.%)	29 (48.3)	0.53
Delivery mode			
*Vaginal delivery*	215 (89.6)	54 (90.0)	0.92
*Caesarean delivery*	25 (10.4)	6 (10.0)	
Delivery by doctor§			
*Yes*	49/237 (20.7)	9/60 (15.0)	0.32
*No*	188/237 (79.3)	51/60 (85.0)	
Delivery by nurse/midwife§			
*Yes*	186/237 (78.5)	53/60 (88.3)	0.09
*No*	51/237 (21.5)	7/60 (11.7)	
Birthweight in grams, mean (SD (range))	2096 (254 (1500, 2490))	2072 (205 (1620, 2400))	0.51
Gestational age at birth in weeks, mean (SD (range))§	35.9 (2.7 (27.3, 43.9))	35.8 (2.3 (30.0, 41.1))	0.79
Term status, n/N (%)			
*Preterm*	125/239 (52.3)	36/60 (60.0)	0.28
*Term*	114/239 (47.7)	24/60 (40.0)	
Low birthweight type, n/N (%)			
*Preterm small for gestational age*	27/239 (11.3)	11/60 (18.3)	0.19
*Preterm appropriate for gestational age*	83/239 (34.7)	24/60 (40.0)	
*Preterm large for gestational age*	15/239 (6.3)	1/60 (1.7)	
*Term small for gestational age*	114/239 (47.7)	24/60 (40.0)	
Birth complications			
*No complications*	86 (35.8)	10 (16.7)	0.01
*Premature birth*	92 (38.3)	27 (45.0)	0.35
*Jaundice*	11 (4.6)	8 (13.3)	0.01
*Congenital malformations*	1 (0.42)	0	0.62
*Fever*	37 (15.4)	2 (3.3)	0.01
*Hypoglycaemia*	1 (0.42)	0	0.62

*Data presented as n (%) unless otherwise specified.

†Born 18 October 2019 to 1 April 2020.

‡Born 2 April 2020 to 29 July 2020.

§N = 299, one missing information on gestational age. N = 297, three missing information on who delivered infant.

Regarding LBW type, 18.4% were preterm-SGA, 31.8% were preterm-AGA, 3.7% were preterm-LGA, and 46.2% were term-SGA infants. More preterm-SGA infants were born during the pandemic period compared to the pre-COVID period (25.0% during COVID-19 vs 16.7% pre-COVID-19). The mean birthweight was 2008g (SD 249). Our cohort consisted of slightly more females (52.0%) than males. Most infants were born via vaginal delivery (89.7%). We observed a decrease in NICU admissions during the pandemic period compared to the pre-pandemic period (28.3% during COVID-19 vs 30.8% pre-COVID-19; *P* = 0.400); this difference also existed between the two facilities, with no reported NICU admissions at Bwaila hospital during COVID-19 period **(****Table 2****)**. We also found differences in separation at birth between mother-infant dyads, with less separation during the pandemic period (81.7% during COVID-19 *vs* 90.7% pre-COVID-19; *P* = 0.046).

**Table 2:** Location of care at birth for infants born before compared to during the COVID-19 pandemic in Malawi*

	Pooled (n = 300)†	Pre-COVID-19 (n = 240)‡	During COVID-19 (n = 60)§	*P*-value
**NICU admission**	n = 300	n = 240	n = 60	
Yes	91 (30.3)	74 (30.8)	17 (28.3)	0.40‖
No	186 (62.0)	143 (59.6)	43 (71.7)	
No data in chart	23 (7.7)	23 (9.6)	0 (0.0)	
**NICU admission at secondary facility**	n = 12	n = 12	n = 0	
Yes	10 (83.3)	10 (83.3)	0 (0.0)	N/A
No	2 (16.7)	2 (16.7)	0 (0.0)	
**NICU admission at tertiary facility**	n = 288	n = 228	n = 60	
Yes	81 (28.1)	64 (28.1)	17 (28.3)	0.67‖
No	184 (63.9)	141 (61.8)	43 (71.7)	
No data in chart	23 (8.0)	23 (10.1)	0	
**Separation at birth**	n = 297	n = 237	n = 60	
Yes	264 (88.9)	215 (90.7)	49 (81.7)	0.05
No	33 (11.1)	22 (9.3)	11 (18.3)	
**Separation at birth at secondary facility**	n = 12	n = 12	n = 0	
Yes	11 (91.7)	11 (91.7)	0 (0.0)	N/A
No	1 (8.3)	1 (8.3)	0 (0.0)	
**Separation at birth at tertiary facility**	n = 288	n = 228	n = 60	
Yes	253 (88.8)	204 (90.7)	49 (81.7)	0.05
No	32 (11.2)	21 (9.3)	11 (18.3)	

NICU – neonatal intensive care unit

*Data are presented as n (%) unless otherwise specified.

†Born 18 October 2019 to 29 July 2020.

‡Born 18 October 2019 to 1 April 2020.

§Born 2 April 2020 to 29 July 2020.

‖NICU admission *P*-value excludes missing data on NICU admission from χ^2^ calculations

### Feeding

At baseline, 24.5% of the infants initiated breastfeeding within one hour of birth, with the median time of initiation of 1.0-hour (interquartile range (IQR) = 0.0-8.0) **(****Table 3****)**. Early initiation of breastfeeding decreased by half during the pandemic period compared to the pre-COVID-19 period (14.6% during COVID-19 vs 27.2% pre-COVID-19; *P* = 0.053). Overall, 86.5% of the mothers reported ever receiving breastfeeding support; most (67.4%) reported being talked to in theory about proper latch/positioning. Healthcare providers (97.7%) offered most of the support. Discussing proper latching/positioning support was significantly reduced during the pandemic period (44.9% during COVID-19 vs 72.7% pre-COVID-19, *P* < 0.001) as was physical support with positioning the baby (14.3% during COVID-19 vs 45.5% pre-COVID-19, *P* < 0.001) during the pandemic (**Table 3**). Most of the infants were exclusively breastfed at baseline and at week one postpartum **(****Table 4****).**

**Table 3:** Feeding indicators for infants born before compared to during the COVID-19 pandemic in Malawi

	Pooled (n = 300)*	Pre-COVID-19 (n = 240)†	During COVID-19 (n = 60)‡	*P*-value
**Initiation of breastfeeding within 1 h, n/N (%)**	64/261 (24.5)	56/206 (27.2)	8/55 (14.5)	0.05
**Timing of initiation of BF, n (median (IQR; range in hours))**	33 (1.0 (0-3.0; 0-8.0))	23 (1.0 (0-3.0; 0-8.0))	10 (2.0, (0-2.0; 0-4.0))	0.39
**Ever expressed breast milk in first 2 weeks, n/N (%)**	216/298 (72.5)§	171/239 (71.6)	45/59 (76.3)	0.46
**Exclusive breastfeeding to 10 weeks, n/N (%)**	187/300 (62.3)	152/240 (63.6)	35/60 (58.3)	0.47
**Ever received lactation support, n (%)**	258 (86.6)‖	209 (87.1)	49 (81.7)	0.21
**Among those who received support at baseline by type of support received, n (%)**				
Talking in theory about proper latch/positioning	174 (67.4)	152 (72.7)	22 (44.9)	<0.001
Support with positioning mom/baby	102 (39.5)	95 (45.5)	7 (14.3)	<0.001
Support with latching	109 (42.3)	92 (44.0)	17 (34.7)	0.23
Support with expressing breastmilk	158 (61.2)	129 (61.7)	29 (59.2)	0.74
Support for feeding with bottle/cup	104 (40.3)	85 (40.7)	19 (38.8)	0.81
Other	51 (19.8)	32 (15.3)	19 (38.8)	<0.001
**Among those who received support at baseline by who provided support, n (%)**				
Healthcare provider	252 (97.7)‖	206 (98.6)	46 (93.9)	0.05
Lactation consultant	4 (1.6)	2 (0.96)	2 (4.1)	0.11
Family member	41 (15.9)	40 (19.1)	1 (2.0)	<0.01

IQR – interquartile range

*Born 18 October 2019 to 29 July 2020.

†Born 18 October 2019 to 1 April 2020.

‡Born 2 April 2020 to 29 July 2020.

§Two missing.

‖42 missing data on feeding counselling.

**Table 4:** Feeding profile at baseline and week 1 for infants born before compared to during the COVID-19 pandemic in Malawi*

	Baseline			Week 1		
	**Pooled (n = 300)†**	**Pre-COVID-19 (n = 240)‡**	**During COVID-19 (n = 60)§**	**Pooled (n = 262)†**	**Pre-COVID-19 (n = 213)‡**	**During COVID-19 (n = 49)§**
Exclusive breastfeeding	292 (97.3)	234 (97.5)	58 (96.7)	258 (98.5)	209 (98.1)	49 (100)
Mixed milk feeding	1 (0.3)	1 (0.4)	0 (0.0)	4 (1.5)	4 (1.9)	0 (0.0)
No breastmilk	0 (0.0)	0 (0.0)	0 (0.0)	0 (0.0)	0 (0.0)	0 (0.0)
No food given yet	7 (2.3)	5 (2.1)	2 (3.3)	0 (0.0)	0 (0.0)	0 (0.0)

*Data presented as n (%).

†Born 18 October 2019 to 29 July 2020.

‡Born 18 October 2019 to 1 April 2020.

§Born 2 April 2020 to 29 July 2020.

### Growth

**Figure 3:**
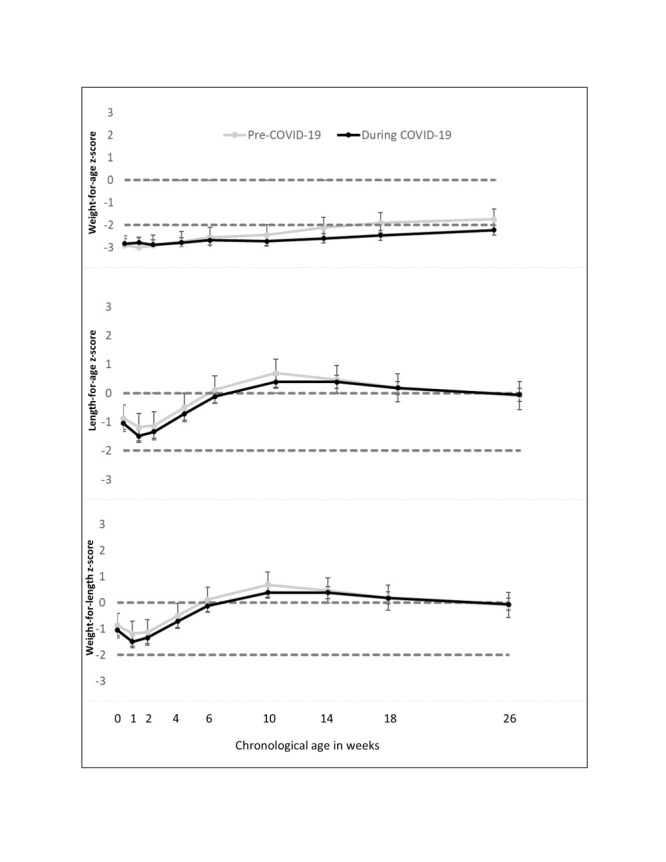
Unadjusted weight-for-age, length-for-age and weight-for-length z-scores by visit week for a cohort of moderately LBW born pre-COVID-19 (before 1 April 2020) and during COVID-19 (on or after 2 April 2020).

Overall, 19.1% (50/263) of infants did not regain their birthweight by two weeks **(****Table 5****)**. In the pooled population, stunting was the most prevalent indicator of poor growth at week 10 (46.3%) followed by underweight (28.9%) and wasting (2.0%). However, we found no statistically significant differences in the growth indicators of stunting, underweight, and wasting seen during the COVID-19 period compared to the pre-COVID-19 period. Regarding growth over time, mean WAZ scores for infants born during COVID-19 did not exceed the reference median at any follow-up time. During the first 10 weeks, infants born prior to the COVID-19 pandemic and those born during the pandemic had similar WAZ, LAZ, and WLZ scores (**Figure 3**).

**Table 5:** Growth indicators for infants born before compared to during the COVID-19 pandemic in Malawi*

	Time period			
	**Pooled†**	**Pre-COVID-19‡**	**During COVID-19§**	***P*-value**
Week 2, n	263	216	47	
No birthweight regained by week 2, n (%)	50 (19.1)	45 (17.2)	5 (1.9)	0.10
Week 10, n	254	127	127	
Weight-for-age Z scores at week 10, mean (SD (range))	-1.0 (1.7 (-5.7, 3.5))	-1.1 (1.8 (-5.7, 3.5))	-0.76 (1.5 (-3.6, 2.3))	0.24
Length-for-age Z scores at week 10, mean (SD (range))	-1.6 (1.7 (-5.7, 3.9))	-1.6 (1.8 (-5.7, 3.9))	-1.6 (1.3 (-4.8, 2.3))	0.78
Weight-for-length Z scores at 10 weeks, mean (SD (range))	0.30 (1.3 (-5.0, 4.4))	0.28 (1.3 (-5.0, 4.4))	0.37 (1.3 (-1.8, 3.5))	0.66
Underweight at 10 weeks, n (%)	73 (28.9)	62 (30.4)	11 (22.5)	0.27
Stunting at 10 weeks, n (%)	117 (46.3)	92 (45.1)	25 (51.0)	0.46
Wasting at 10 weeks, n (%)	5 (2.0)	5 (2.5)	0 (0)	0.27

SD – standard deviation

*For frequencies n = 253, one infant missing anthropometric data at 10 weeks.

†Born 18 October 2019 to 29 July 2020.

‡Born 18 October 2019 to 1 April 2020.

§Born 2 April 2020 to 29 July 2020.

### Morbidity and mortality outcomes

Overall, 12.9% of the infants experienced overnight hospital admission between baseline and week 10, with less overnight admissions observed during COVID-19 compared to pre-COVID-19 (5.5% during COVID-19 *vs* 14.3% pre-COVID-19; *P* = 0.066) **(****Table 6****).** There was less reported cough and difficulty breathing, fast breathing, and/or chest in-drawing symptoms for infants during compared to before the pandemic (47.3% during COVID-19 *vs* 62.5% pre-COVID-19; *P* = 0.039). The overall mortality for the enrolled and analysed cohort was 4.0% (n = 12/300), with slightly more deaths observed among infants born before the pandemic (3.3% during COVID-19 vs 4.2% pre-COVID*-19*, *P* = 0.170).

**Table 6:** Morbidity and mortality indicators of interest and relevant time periods*

	Pooled (n = 279)†	Pre-COVID-19 (n = 224)‡	During COVID-19 (n = 55)§	*P*-value
Ever overnight admission between baseline and week 10 (inclusive)	36 (12.9)	33 (14.7)	3 (5.5)	0.07
Ever visited hospital due to diarrhoea	111 (39.8)	87 (38.8)	24 (43.6)	0.51
Ever had cough and difficulty breathing, fast breathing and/or chest indrawing	166 (59.5)	140 (62.5)	26 (47.3)	0.04
Ever had fever	174 (62.4)	138 (61.6)	36 (35.5)	0.60
Ever vomited	84 (30.1)	70 (31.3)	14 (25.5)	0.40
Ever had malaria	12 (4.3)	12 (5.4)	0	0.08
Infants with sick visits, n	100	98	2	
Sick visit infant hospitalisations, n/N (%)	6/100 (6.0)	6/98 (6.1)	0/2 (0)	0.72
Infants with data on duration of hospitalisation from sick visits, n	4	4	0	
Duration of hospitalisation from sick visits, mean (SD (range in days))	3.8 (2.1 (2.0-6.0))	3.8 (2.1 (2.0-6.0))	-	-
Mortality, n/N (%)	12/300 (4.0)	10/240 (4.2)	2/60 (3.3)	0.17

SD – standard deviation

*Data are presented as n (%) unless otherwise specified.

†Born 18 October 2019 to 29 July 2020.

‡Born 18 October 2019 to 1 April 2020.

§Born 2 April 2020 to 29 July 2020.

## DISCUSSION

Here we described the changes in complications, care, feeding, growth, and health outcomes among moderately LBW infants born and followed up before compared to during the COVID-19 pandemic in Malawi and highlighted its impact on early infant feeding and lactation support in two public hospitals. Among this cohort of moderately LBW infants, the COVID-19 pandemic had an impact on health services and feeding practices. The COVID-19 period was associated with a delay in the early initiation of breastfeeding, with an increase in breastfeeding initiated more than one hour from birth likely due to shortages of nurses to take the baby to the mother in time. The WHO recommends early initiation of breastfeeding within one hour of birth [24] and continued to do so following the pandemic outbreak, even for mothers who were confirmed or suspected COVID-19 cases [25]. Early initiation of breastfeeding is crucial for establishing breastfeeding, prevention of infections, optimal early growth, and reduction of newborn mortality [26-29]. Additionally, delays in initiation of breastfeeding may exacerbate respiratory outcomes. One study in Nepal demonstrated that a delay in breastfeeding initiation during the COVID-19 pandemic was associated with an increased risk of acute respiratory infection in children under two years of age [30].

Lactation support and management provided were also impacted by the pandemic, with decreased discussion of latch/positioning and physical support provided, possibly contributing to an increase in delayed initiation of breastfeeding during the pandemic period. These findings indicate that, even though they are considered essential core health services [31], maternal and newborn health services reduced due to COVID-19 pandemic in LMICs. The quality of care deteriorated, risking deaths and reversals of all the gains over the past two decades [32]. Similar findings have been reported elsewhere [9,15,33].

There was also an increase in the number of infants born with birth complications during the pandemic, the most notable being neonatal jaundice. Interestingly, an increase in neonatal jaundice during the pandemic period has also been reported in a large study in China [34]. It is most commonly caused by physiological reasons, mainly maternal-infant blood incompatibility and immaturity of the infant`s liver in handling haemolysis [35]. LBW babies are at increased risk of neonatal jaundice, which also may be caused by neonatal sepsis [36,37]. Breastfeeding jaundice, an exaggerated form of physiologic jaundice which occurs when the baby is fed inadequate milk, may have occurred in the context of this cohort [38], possibly due to delayed initiation of breastfeeding and reduced lactation support.

We observed less NICU admissions during the pandemic highlighting a possible reduction in LBW infants born in these hospitals requiring NICU care or reduced capacities of the hospital units to provide high level care during the pandemic; although we cannot assign causality, this is suggestive that accessing healthcare for issues that were not COVID-19 related might have been impacted. A study of 16 640 infants in China found a significant decline in the neonatal hospital admissions during a COVID-19 impacted time period compared to pre-COVID-19 [33]. Other evidence shows that the number of patients attending essential healthcare services declined during the pandemic [15,39-41]. However, the change in health seeking behaviour did not affect ANC visits among the women in our study, highlighting that the pregnant mothers likely valued these visits. These findings differ from those of a study done in Saudi Arabia, where approximately a third of women missed their ANC visits for fear of the COVID-19 infection [42]. However, mother-infant dyads who were enrolled and followed up during the pandemic in our study may have still had ANC visits that occurred before the pandemic.

We noted lower mother-infant dyad separation rates during the COVID-19 compared to the pre-COVID-19 period. These findings are in contrast to other studies that reported high rates of mother-infant separation as a result of the pandemic [39,43,44]. There was also no clear effect on infant growth parameters except for stunting, which may reflect longer term effects on growth outcomes, possibly due to a short follow-up period in this study.

### Strengths and limitations

Some study strengths are the prospective data collection from both a tertiary and secondary care facility, utilisation of both maternal reports and observations, and few losses to follow-up. This analysis also reflects how pandemics or other similar events that may disrupt health systems may adversely affect the well-being of vulnerable infants, informing future readiness and resilience building. However, the study had some limitations. We followed up a relatively small sample size of infants born during the pandemic period and the study follow-up period was short, limiting the strength of our analysis on the impact of the pandemic on the morbidity and health outcomes due to the pandemic, and the generalisability of our findings. We may not have had sufficient power to detect statistical differences. Our analyses were intended to be descriptive, so we have limited our modelling. As such, statistically significant associations should be interpreted with caution, as we could not control for confounding. Second, we did not collect data on confirmed COVID-19 infection for neither mothers nor infants during the study follow-up period.

## CONCLUSIONS

Our findings highlight the need to optimise early initiation of breastfeeding and lactation support for infants during this and future pandemics, or any events potentially disrupting health delivery systems. There is a need for larger multicentre studies to evaluate the long-term outcomes of moderately LBW infants born during the COVID-19 pandemic, including growth outcomes and determine the impact of pandemic restrictive measures on access to lactation support and counselling and promotion of early initiation of breastfeeding.
